# Supplementary material for: Analysis of PPARγ Signaling Activity in Psoriasis
Source: Int J Mol Sci. 2021 Aug 10;22(16):8603. doi: 10.3390/ijms22168603 (PMC8395241; doi:10.3390/ijms22168603)
Supplement: Supplementary file 1 [file ijms-22-08603-s001.zip › Supplemental materials_Analysis of PPARg signaling activity in psoriasis/Pathway models/Models images and html files/PPARG inhibits phenotypes assotiated with psoriasis/36348.html]

vasculitis


# Disease vasculitis

|  |  |
| --- | --- |
| URN | urn:agi-meshdis:Vasculitis |
| Total Entities | 29 |
| Connectivity | 3865 |
| Name | vasculitis |

---

|  |  |
| --- | --- |
| ChildConcepts | hemorrhagic vasculitis |
|  | central nervous system vasculitis |
|  | phlebitis |
|  | cerebral vasculitis |
|  | granulomatous vasculitis |
|  | arteritis |
|  | ANCA-associated vasculitis |
|  | microscopic polyangiitis |
|  | systemic vasculitis |
|  | rheumatoid vasculitis |
|  | capillaritis |
|  | pulmonary vasculitis |
|  | small vessel vasculitis |
|  | mixed cryoglobulinemia |
|  | vasculitic rash |
|  | livedo racemosa |
|  | livedo vasculitis |
|  | livedoid vasculopathy |
|  | Henoch-Schonlein purpura |
|  | Churg-Strauss syndrome |
|  | leukocytoclastic vasculitis |
|  | cutaneous vasculitis |
|  | Goodpasture's syndrome |
|  | retinal vasculitis |
|  | large vessel vasculitis |
|  | obliterating vasculitis |
|  | thromboangiitis obliterans |
|  | hypocomplementemic urticarial vasculitis |
|  | cryoglobulinemic vasculitis |

---

|  |  |
| --- | --- |
| Pathway | PPARG inhibits phenotypes assotiated with psoriasis |
|  | Neighbors of vasculitis |

---

|  |  |
| --- | --- |
| MedScan ID | 9004351 |

---

|  |  |
| --- | --- |
| Alias | vascular inflammations |
|  | angiitis |
|  | Vasculitides |
|  | Vasculitis |
|  | vasculitic lesion |
|  | vasculitic lesions |
|  | acute vasculitis |
|  | angiitides |
|  | vascular inflammation |
|  | obliterating vasculitis |
|  | angitis |

---

|  |  |
| --- | --- |
| MeSH ID | D014657 |
|  | M0022540 |

---

|  |  |
| --- | --- |
| MeSH Heading | Vasculitis |

---
